# Recurrent temporal networks and language acquisition—from corticostriatal neurophysiology to reservoir computing

**DOI:** 10.3389/fpsyg.2013.00500

**Published:** 2013-08-05

**Authors:** Peter F. Dominey

**Affiliations:** Robot Cognition Laboratory, Centre National de la Recherche Scientifique and INSERM Stem Cell and Brain Research InstituteBron Cedex, France

**Keywords:** reservoir computing, recurrent network, P600, grammatical construction, striatum

## Abstract

One of the most paradoxical aspects of human language is that it is so unlike any other form of behavior in the animal world, yet at the same time, it has developed in a species that is not far removed from ancestral species that do not possess language. While aspects of non-human primate and avian interaction clearly constitute communication, this communication appears distinct from the rich, combinatorial and abstract quality of human language. So how does the human primate brain allow for language? In an effort to answer this question, a line of research has been developed that attempts to build a language processing capability based in part on the gross neuroanatomy of the corticostriatal system of the human brain. This paper situates this research program in its historical context, that begins with the primate oculomotor system and sensorimotor sequencing, and passes, via recent advances in reservoir computing to provide insight into the open questions, and possible approaches, for future research that attempts to model language processing. One novel and useful idea from this research is that the overlap of cortical projections onto common regions in the striatum allows for adaptive binding of cortical signals from distinct circuits, under the control of dopamine, which has a strong adaptive advantage. A second idea is that recurrent cortical networks with fixed connections can represent arbitrary sequential and temporal structure, which is the basis of the reservoir computing framework. Finally, bringing these notions together, a relatively simple mechanism can be built for learning the grammatical constructions, as the mappings from surface structure of sentences to their meaning. This research suggests that the components of language that link conceptual structure to grammatical structure may be much simpler that has been proposed in other research programs. It also suggests that part of the residual complexity is in the conceptual system itself.

## Introduction

We begin with the neurophysiological basis of orienting behavior, which provides the framework that leads to language. In a dynamic and changing world, filled with predators and prey, the ability to rapidly orient one's spatial attention to the right place is a question of survival. In mammals with mobile heads (e.g., cats) and primates with mobile eyes (monkeys and man), the ability to orient allows these animals to control their attention to the environment with high precision, and with a temporal reactivity on the scale of hundreds of milliseconds—fractions of a second. In the 1980's research in the oculomotor system of the cat and macaque monkey reached a certain height of completion, and the neural circuits that processed information from visual input to motor response were specified at a fairly high level of detail [reviewed in Dominey and Arbib (1992)]. This represented an important phase in cognitive neuroscience, because the same circuits that specified motor control and spatial attention in the oculomotor system were templates for parallel circuits that would provide part of the basis for higher cognitive function and language.

**Figure 1:**
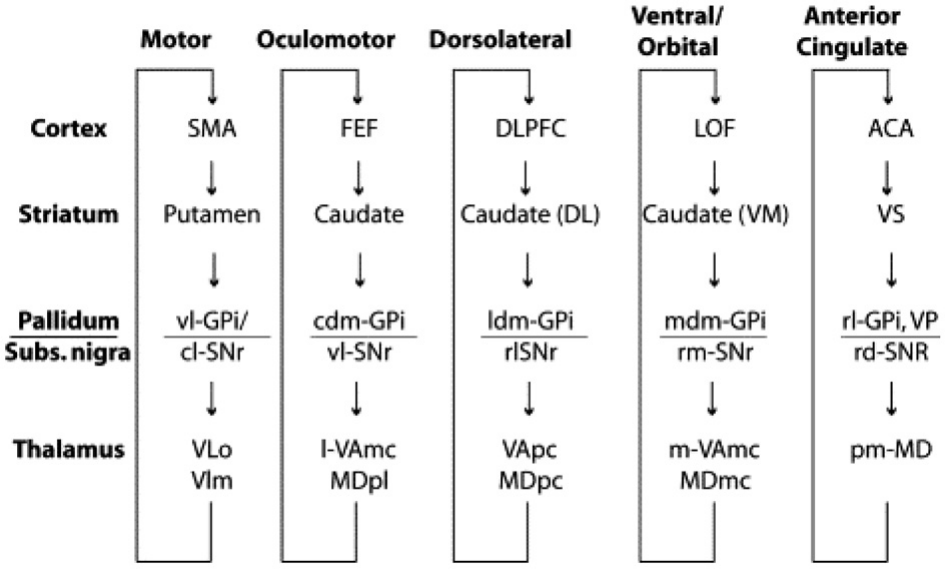
**Parallel organization of the cortico-striato-thalamo-cortical loops.** From Grahn et al. (2009), modified from Alexander et al. (1986).

In this context, one of the major architectural properties of the primate brain is the massive organized projection from cortex to striatum (Selemon and Goldman-Rakic, 1985; Lehericy et al., 2004). Essentially all of neocortex projects in a topographically organized manner to the striatum, through the pallidum to the thalamus and back to cortex (Ilinsky et al., 1985), thus yielding what can be considered as a set of largely distinct and segregated corticostriatal circuits or loops (see Figure 1), dedicated to distinct functions, including control of different motor systems such as the oculomotor system, and the limbic reward system (Alexander et al., 1986). This paper will argue that this notion can be extended to a cortico-striatal language circuit (Dominey and Inui, 2009; Dominey et al., 2009).

The closed loop structure provides a feedback of the results of the outcome of the system back into cortex. Such feedback connections have been demonstrated to play an important role in memory and sequence processing (Jaeger and Haas, 2004; Jaeger et al., 2007).

At the same time that the functional neuroanatomy of the oculomotor loop had been quite well characterized and modeled in a neurophysiologically realistic manner (Dominey and Arbib, 1992), the mechanisms for dopamine modulated plasticity in the corticostriatal synapse (Calabresi et al., 1992) that could lead to adaptive behavior were also being characterized (Robbins et al., 1990; Reading et al., 1991). For example, Reading and Robins demonstrated how the lateral caudate-putamen is required for the learning of arbitrary stimulus-response associations (Reading et al., 1991), which were also impaired in the absence of cortico-striatal dopamine (Robbins et al., 1990).

This inspired us to consider that the cortico-striatal junction could be used as a convergence point where information from different modalities could be functionally linked by dopamine-modulated cortico-striatal plasticity (Dominey et al., 1995). Indeed, while the “central dogma” of the corticostriatal system presents a parallel and segregated set of loops as illustrated in Figure 1, from the beginning this was known to be a simplification (Selemon and Goldman-Rakic, 1985), as in fact, the projections from cortex to striatum display a more complex topography. While the main and most dense projections follow the parallel circuit concept, more diffuse projections form larger territories, leading to large overlap of the different circuits (Selemon and Goldman-Rakic, 1985). These overlaps provide a crucial function—they allow the adaptive binding together of cortical signals from different functional circuits. Thus, for example, visual features from infero-temporal (IT) cortex can become linked to direction eye movements (saccades) to different locations in space. We modeled this framework by extending the oculomotor model so that the oculomotor region of the caudate received inputs from the oculomotor frontal eye fields, consistent with the parallel circuits in Figure 1, and in addition it received projections from the inferior temporal cortex, consistent with known neuroanatomy (Selemon and Goldman-Rakic, 1985), which code the features of visual stimuli. The resulting model provided the first account of how reward-related dopamine could strengthen corticostriatal synapses binding stimulus coding to behavioral response coding (Dominey et al., 1995). The relevance of this historical interlude into the functional neuroanatomy of the corticostriatal system will soon become apparent, as we make the link from associative learning, to sequence learning to language.

## Serial, temporal and abstract structure and the initial state

Twenty-five years ago, Barone and Joseph (1989) studied neural activity in the prefrontal cortex (PFC) of monkeys that had been trained to perform a simple task that involved watching the presentation of a visual sequence on a response button board, and then after a short delay, reproducing the sequence by touching the buttons on the board in the same order that they were presented. They observed that neurons in the dorsolateral prefrontal cortex (DLPFC) displayed two characteristic responses to stimuli in the sequence task. First, as had previously been observed, the neurons were spatially selective, with preferences for stimuli in particular locations in the retinal image. The second characteristic was new, and revolutionary: many of these neurons also displayed a “sequence rank” effect, that is, they had preferences for stimuli that had appeared first, second or last in the input sequence. Thus, the spatial selectivity in many neurons was modulated by the rank or order of the element in the sequence. This indicated that DLPFC embodies a mechanism for discriminating the order of items in a perceptual sequence.

In an effort to understand how this order-sensitive property could result from principal characteristics of the PFC, we recalled that a second major architectural property of the primate brain (the first being the massive cortico-striatal projection) is the abundance of local connectivity in cortico-cortical connections, or recurrent connections, particularly in the frontal cortex (Goldman-Rakic, 1987). Recurrent connections in neural networks provide known dynamical system properties, and indeed in the context of Elman's simple recurrent network (SRN) the power of recurrent connections in language-related processing was revealed (Elman, 1990, 1991). Intuitively, recurrent connections allow information from past events to remain coded, circulating through these connections, and thus allowing the past to influence the coding of new inputs. This provides an intrinsic sequence coding capability.

The use of recurrent connections in the context of synaptic adaptation also unveiled the immense technical challenge of determining how a given recurrent connection contributed to error in the network response, since over multiple time steps the state of activation in the recurrent network changes dynamically (Pearlmutter, 1995). The solution developed by Elman was to limit the simulation of cycles through the recurrent net to one or two time steps. This provided a dramatic simplification of the learning algorithm while preserving the essential property of recurrent connections. This introduced a significant limitation, however, with respect to the processing of time.

A principal objective of computational neuroscience is to simulate and explain neural activity over the time course of the behavioral experiment. Thus, in Barone and Joseph's sequenceing task, stimuli are presented for a certain duration, and the subsequent execution of the sequence by the animal unfolds in time, including the reaction times for the individual responses. The simplification in the SRN renders such realistic treatment of time impossible, as the time between successive sequence elements is fixed by the learning algorithm.

In order to circumvent the technical challenges of recurrent learning in time, we chose an alternative approach. We decided to fix the connection strengths of the recurrent connections at the outset so as to provide the simulated PFC network with a dynamic structure that would retain a trace of previous inputs via the recurrent connections. The resulting patterns of activity in the cortical network could then be associated with the corresponding behavioral outputs by reward-related (dopaminergic) plasticity in the corticostriatal synapses (Dominey, 1995; Dominey et al., 1995).

The resulting system is illustrated in Figure 2. The principal characteristics are the presence of fixed recurrent connections in the PFC network (corresponding to the DLPFC in Figure 1), and modifiable connections between these PFC neurons and the neurons in the striatum (caudate nucleus—CD), which form an associative memory, associating dynamic states in the recurrent network with the desired output response. It is noteworthy that this combination of fixed recurrent connections, and modifiable connections to “readout” neurons was the first characterization of what has now come to be known as the reservoir principle in reservoir computing (Maass et al., 2002; Lukosevicius and Jaeger, 2009). The resulting network displayed a number of interesting properties.

**Figure 2:**
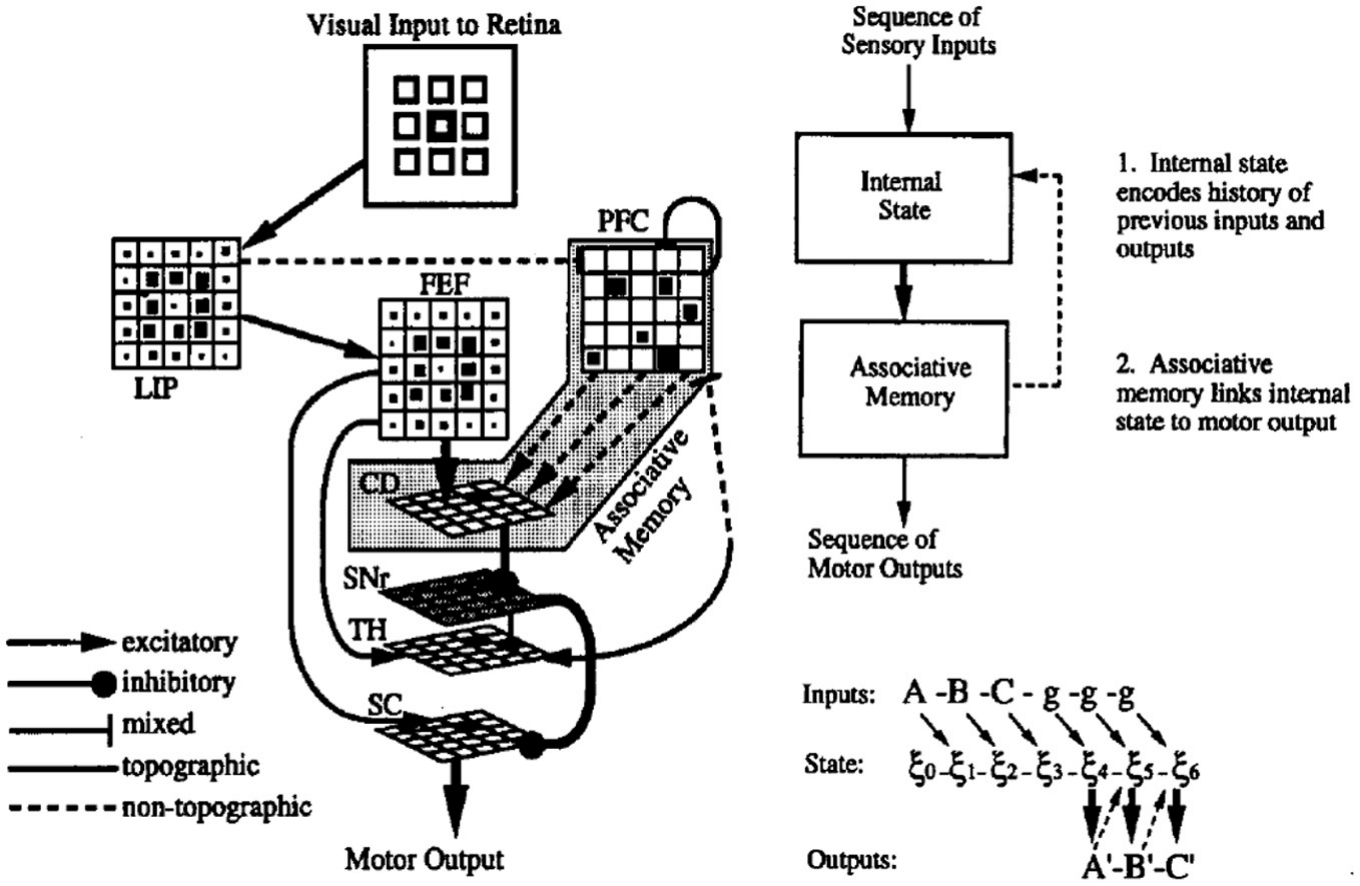
**Model of cortico-striatal system for sensorimotor sequence learning. Left**—neuroanatomical structure of model. Visual input to simulated retina projects to lateral interparietal cortex (LIP) and frontal fields (FEF), and prefrontal cortex (PFC) (via mixed connections). PFC has recurrent connections, rendering it a rich dynamical system, and projects with modifiable connections to the caudate nucleus of the striatum (CD), which serves to activate the motor superior colliculus (SC) via the oculomotor circuit. **Right**—synthetic view. Recurrent PFC network encodes internal state, and projects with modifiable connects to associative memory. Feedback connections from associative memory to internal state allow state to be influenced by the results of the learned associations. From Dominey (1995).

First, it was able to explain the behavior of monkeys in the Barone and Joseph sequence learning task, and more interestingly, the neural activity in simulated PFC neurons displayed the same combination of spatial and sequence rank coding properties as those recorded in the monkey PFC (Dominey et al., 1995). In particular, the simulated PFC neurons were spatially selective, and this spatial selectivity was modulated by the rank of the spatial target in the sequence. This was a computational neuroscience success.

Second, the model displayed a robust sequence learning capability. Figure 3 illustrates the dynamic activity within PFC neurons during the presentation and replay of a 25 element sequence. One can observe that the pattern of activation in PFC (recurrent network) neurons displays a rich dynamic behavior, and that indeed, the states in PFC corresponding to the different elements in the sequence are indeed separable, as revealed by the observation that the cosines of the state vectors are never equal to unity (i.e., the state vectors are never identical). In this context, the model could account for (Dominey, 1998a) and predict (Dominey, 1998b) human sequence learning behavior in the well studied serial reaction time (SRT) task. Because the connections from cortex to striatum are modified by learning, neurons in the striatum become activated with reduced reaction times in cases where learning is significant. That is, when responding to visual inputs presented in a well-learned sequence, stronger learned cortico-striatal connections lead to faster activation of the striatal response neurons, leading to a reduced number of simulation time steps for generating the model output. For elements presented in a random sequence, there was no learning effect, and significantly more time steps were required to generate the response in the striatal neurons. Details can be found in Dominey (1998a,b).

**Figure 3:**
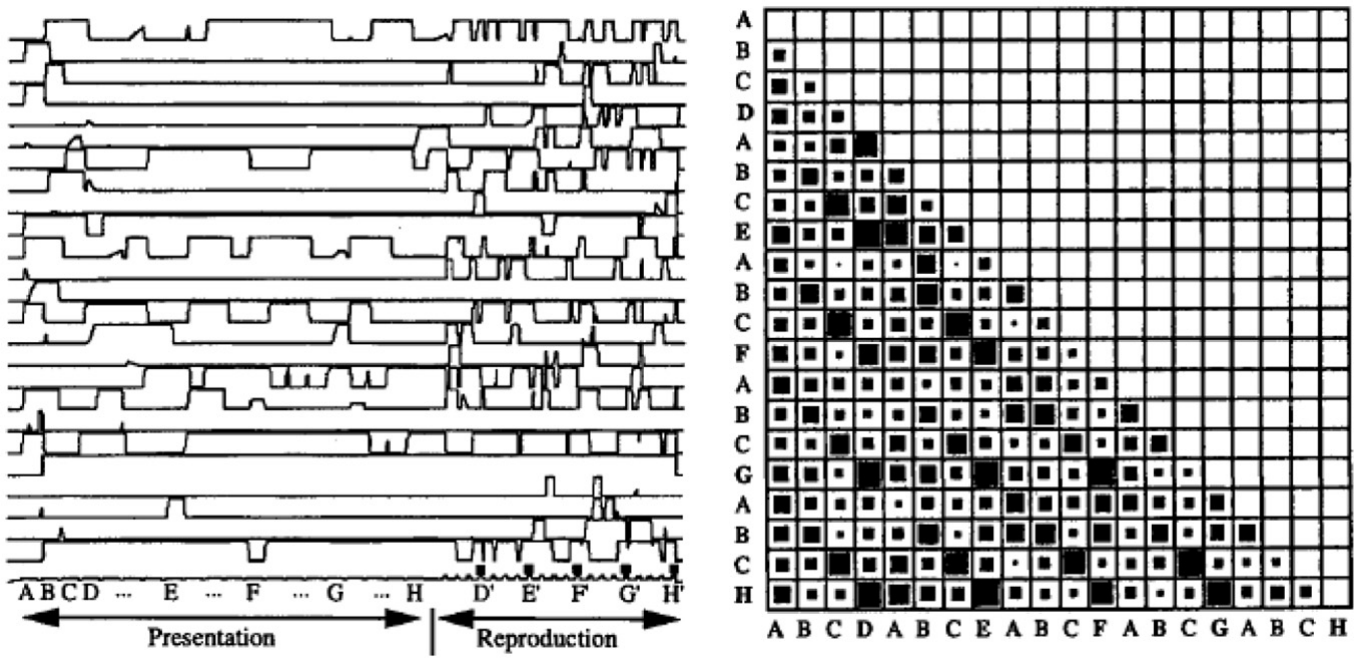
**Neural activity during complex sequence processing. Left**—time trace of activity in 25 PFC neurons during presentation and subsequent replay of a complex sequence of order 4. **Right**—vector cosines of PFC state vector activity during the response choice for each of the 25 responses in the output sequence execution. Cosine represented spatially with 0 as empty and 1 as fully filled case. Note that the cases (and subsequent diagonals in the matrix) corresponding to choices of D, E, F, G, and H have relatively high cosines, indicating that the PFC states are similar, but not identical, for these elements. From Dominey (1995).

While the model thus provided a robust learning for serial and temporal structure, it failed to learn what we called abstract structure. Serial and abstract structure are defined such that the two sequences ABCBAC and DEFEDF have different serial structure (i.e., the serial order of specific sequence elements), but identical abstract structure (i.e., the relations between repeating elements within the sequence), following an internal repetitive pattern 123213 (Dominey et al., 1998). In order to account for learning such abstract structure, a system would need additional processing mechanisms in order to detect that the current element in a sequence is a repetition of an earlier element, and then to “recode” the sequence in terms of this pattern of repeating elements (Dominey et al., 1998).

We introduced these modifications (Dominey et al., 1998), and the resulting hybrid model, illustrated in Figure 4, could thus learn serial, temporal, and abstract structure of perceptual-motor sequences. To demonstrate the importance of this system in helping to characterize the human initial state in language learning, we chose three landmark papers that defined infants' abilities to implicitly learn the serial (Saffran et al., 1996), temporal (Nazzi et al., 1998), and abstract structure (Marcus et al., 1999) of sound sequences. Saffran et al. demonstrated that in minutes infants could learn the sequential structure of syllable sequences, and detect new sequences of the same syllables that violated the learned structure (Saffran et al., 1996). Nazzi et al. similarly demonstrated that infants are sensitive to the rhythmic structure (stress-timed, syllable-timed, and mora-timed) of language stimuli, and can learn to discriminate between distinct classes in minutes (Nazzi et al., 1998). Finally, Marcus et al. demonstrated that infants can just as quickly learn to discriminate abstract structures of syllable sequences like ABA vs. ABB, where A and B represent variables that can be filled in by new syllables (Marcus et al., 1999). That is, the children could recognize a totally new sequence with syllables that they had never heard (i.e., from a new domain) as fitting with the learned rule ABA or ABB. This was an important finding as it indicates infants can generalize over variables in these sequences. These authors argued that the innate ability to discriminate serial, temporal and abstract structure could contribute to the initial state in language learning.

**Figure 4:**
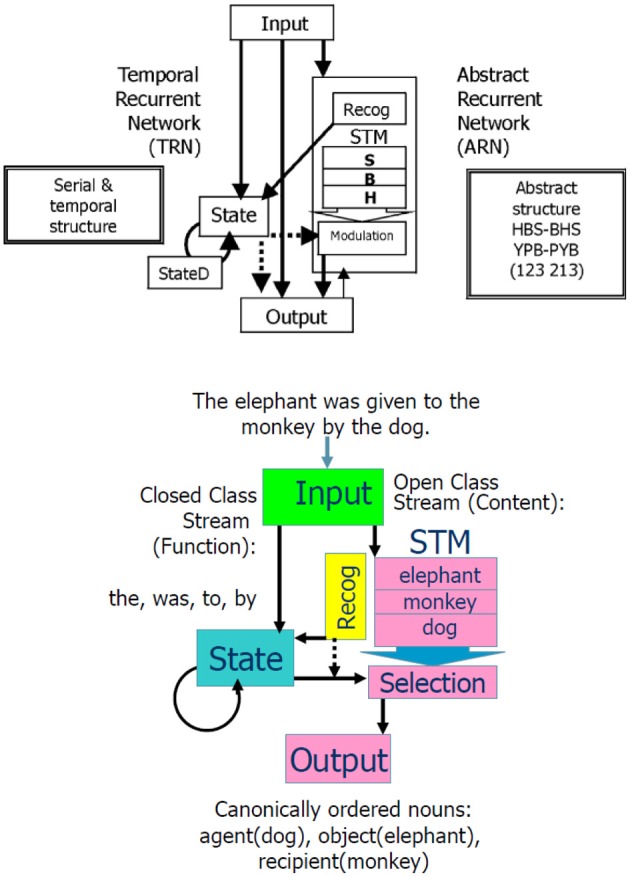
**Combined Abstract Temporal Recurrent Network (ATRN) model. Above:** The temporal recurrent network (TRN) exploits recurrent network dynamics in the recurrently connected neurons in State and StateD layers, to encode serial and temporal structure. To encode the abstract structure common to “isomorphic” sequences such as HBSBHS and YPBPYB, the abstract recurrent network (ARN) stores the N previous elements of the current sequence in a short term memory (STM). The Recognition function compares the current sequence element response generated in Output to the previous elements coded in STM to detect the abstract repetitive structure. This abstracted coding is represented in the recurrent State. In the learned expression of abstract structure knowledge, the contents of STM are selectively provided to the output stream by the activation of Modulation neurons by State neurons. From Dominey et al. (2003). **Below:** Illustration of how the model accommodates language input.

In a series of simulation studies, we replicated these human demonstrations of learning serial (Saffran et al., 1996), temporal (Nazzi et al., 1998), and abstract structure (Marcus et al., 1999) of sound sequences in the hybrid model. Serial and temporal structure were learned by the simpler temporal recurrent network (TRN), and the abstract structure was learned by the abstract recurrent network (ARN) which required a working memory and recognition capability to detect and represent the repetitive structure of the abstract sequences (Dominey and Ramus, 2000). This was an important step in the developing argument about the possible neural mechanisms of language learning.

Subsequent research has suggested that children in the Saffran task may have been picking up on unintended cues related to chunk strength (Perruchet and Pacton, 2006). The TRN has the property that previous inputs influence the state of the recurrent network and thus influence how subsequent input will be processed. Any kind of sequential structure that can be expressed in these terms should lead to learning effects in the TRN. Similarly, Marcus et al.'s (1999) claim that SRN-like models cannot account for their abstract sequence learning results has been challenged. In the Dienes SRN-based model (Dienes et al., 1999), an additional layer was added to allow the mapping of the new domain onto the learned domain, and multiple presentations of the novel stimuli (for adaptation) are required. Likewise, Chang (2002) demonstrated that the standard SRN fails to generalize on an identity construction (related to the ABA construction of Marcus), while his dual path model successfully generalizes. From this perspective it appears that without additional task specific adaptations, Marcus's claim remains intact.

## From sequence learning to language—grammatical construction learning

The notion of abstract rule-based structure suggested a possible link to language processing. In order to test the model in a language processing task, we identified a task that had been developed by Caplan et al. (1985) in which aphasic subjects listened to sentences and then had to indicate the corresponding meaning by pointing to images depicting the agent, object and recipient (i.e., who, gave what to whom), always in that “canonical” order. Thus, in the formal task description the “input sequence” is the sequence of words in the sentence, and the “output sequence” is the sequence agent, object, and recipient, corresponding to the meaning in terms of thematic role assignment. The only cues available for determining “who did what to whom” were the word order and grammatical marking, so this is considered a task of syntactic comprehension.

This approach is consistent with the cue competition model of language (Li and Macwhinney, 2013), which holds that across languages, a limited set of cues including the configuration of grammatical function words (closed class morphology in general), word order and prosody are used to encode the grammatical structure that allows thematic role assignment to take place. We thus implement the cue competition hypothesis (Bates et al., 1982, 1991) focusing on word order and grammatical morphology. In our modeling, the notion is that the sequence of closed class words forms a pattern of activity within the recurrent network, and that this pattern can be associated with the corresponding thematic role specification.

In performing the Caplan task, when faced with an example sentence: “The elephant was given to the monkey by the rabbit,” after hearing this sentence, the experimental subject was required to indicate the meaning by pointing to images depicting the rabbit, elephant, and monkey (corresponding to agent, object, recipient) in that order. Thus, the Caplan task identifies an excellent challenge for language modeling: Given an input sentence, generate a standardized representation of the meaning (i.e., identify the agent, object, and recipient, always in that “canonical” order). The question now is—how can we reformulate this task so as to be processed by our abstract sequencing model. The general notion is that sentence type should correspond to abstract structure. So the Caplan task involves learning nine different abstract structures. Considering our example sentence, if we replace the words with symbols then this becomes an abstract sequence task, where the input is of the form: a E b c d a M e a R, and the corresponding output is R E M (for rabbit, elephant monkey), where upper case letters indicate nouns, and lower class elements indicate all other lexical categories.

We imposed a lexical categorization process at the level of the input processing, with open class words going to the ARN and closed class words going to the TRN, illustrated in the lower panel of Figure 4. Interestingly across languages, these lexical categories tend to have acoustic and distributional signatures that can be used by infants to perform lexical categorization in a process of prosodic bootstrapping (Morgan and Demuth, 1996). Connectionist models have been shown to be able to learn to distinguish open and closed class words from distributional regularities (e.g., Elman, 1990; Chang, 2002). We observed that for French and English, the TRN could encode differences in the prosodic structure of open vs. closed class words in order to perform the lexical categorization between these word classes (Blanc et al., 2003). This provides a demonstration of self-coherence of language in that the most crucial and basic information (i.e., lexical category) is coded at or near the perceptual level.

We thus performed this conversion of the nine sentence types to these abstract sequences. Following the Caplan protocol, five distinct sentences were generated for each sentence type, by replacing the nouns with new nouns. During training, the input sentence was presented to the model, and then in continuation the correctly ordered nouns were presented (i.e., in the agent, object, recipient order). As illustrated in Figure 4, the open class words stored in the STM during the sentence input were then compared with the “response” open class elements. This comparison allowed the sequence of correctly ordered nouns to be “recoded” in terms of their respective matching with the nouns stored in the STM. This recoding became the abstract structure that was learned. That is, for each of the nine sentence types, the model learned the reordering of the nouns from their input order in the sentence, to the output “canonical” order agent, object, recipient. Thus, after training the model could be exposed to a new sentence (with new nouns) that was legal with respect to the learned sentence forms, and it could correctly process the new sentence (reordering the nouns in the agent, object, and recipient order).

## Neural implementation of grammatical constructions

Looking at the model in Figure 4, there is nothing “language specific” about it. Indeed, we proposed that this same model can be used to process abstract sequences, and sentences. This lead to the “audacious” proposition of an “equivalence hypothesis” (Dominey et al., 2003; Dominey, 2005) stating that a common neural system would participate in aspects of processing sentences and abstract non-linguistic sequences. We found strong correlational support for this hypothesis, observing that in agrammatic aphasic patients with left peri-sylivan (Broca's region) lesions, there was a significant correlation between performance in the nine sentence-type Caplan task of syntactic comprehension (described above and modeled), and a task of abstract sequence processing (Dominey et al., 2003). In a further test of this hypothesis we determined whether the left anterior negativity (LAN), an ERP component related to morphosyntactic processing that can reliably be elicited around 350–500 ms after grammatical functions words (Brown et al., 1999) could be elicited by the grammatical “function symbols” in our non-linguistic sequences. Subjects processed sequences with the abstract structures ABC*x*BAC and ABC*y*ABC where x and y, respectively indicated the non-canonical (complex) vs. canonical (simple) rule. We observed a LAN effect for the function symbol which signaled the more complex structure mapping (Hoen and Dominey, 2000), consistent with data from sentence processing. The link between abstract structure and grammatical structure was further revealed when we demonstrated that agrammatic aphasics trained on an abstract structure that corresponds to the remapping of a relativized structure to a canonical structure demonstrated post-test improvement in sentence processing that was specific to the relativized sentences (Hoen et al., 2003). Continuing to test the equivalence hypothesis, we subsequently examined brain activity during sentence and abstract sequence processing with fMRI, and revealed a common network including the dorsal pars opercularis territory of Broca's area for sentence and abstract sequence processing, with additional activation of the ventral pars triangularis region of Broca's area only for sentence processing (Hoen et al., 2006). Thus, the fMRI results confirmed the model's prediction that a common brain system would account for the structural remapping processing aspects of sentence and abstract sequence processing.

Interestingly, this neural computational mechanism appeared capable of providing a neurophysiological grounding of the notion of grammatical construction processing. In this framework, language is considered as a structured inventory of mappings between sentence surface structure and meanings, referred to as grammatical constructions (Goldberg, 1995; Tomasello, 2003). If grammatical constructions are mappings from sentence structure to meaning, then the language system must be able to (a) identify the construction type for a given sentence, and (b) use this information to extract the meaning from the sentence, based on the identified construction. Our thematic role assignment model implements this notion of grammatical constructions. Word order and closed class structure are integrated in the recurrent network, satisfying (a) and this integrated representation is associated, by learning, with the appropriate mapping of open class elements onto their roles in the sentence, satisfying (b). This integration of word order and closed class structure corresponds to an implementation of the cue competition model. We demonstrated the robustness of this model, and provided further support for the cue competition model by testing the neural model with three distinct languages—English, French and Japanese, each with a distinct set of relevant cues. In each of these languages, a universal property holds—the mapping of sentence to meaning is fully specified by the pattern of open and closed class words unique to each grammatical construction type. The model was thus able to learn 38 distinct English constructions, 26 Japanese constructions, and nine French constructions based on the Caplan task. The model thus integrated results from human neurophysiology and behavior into a coherent framework, with a cross-linguistic validation.

**Figure 5:**
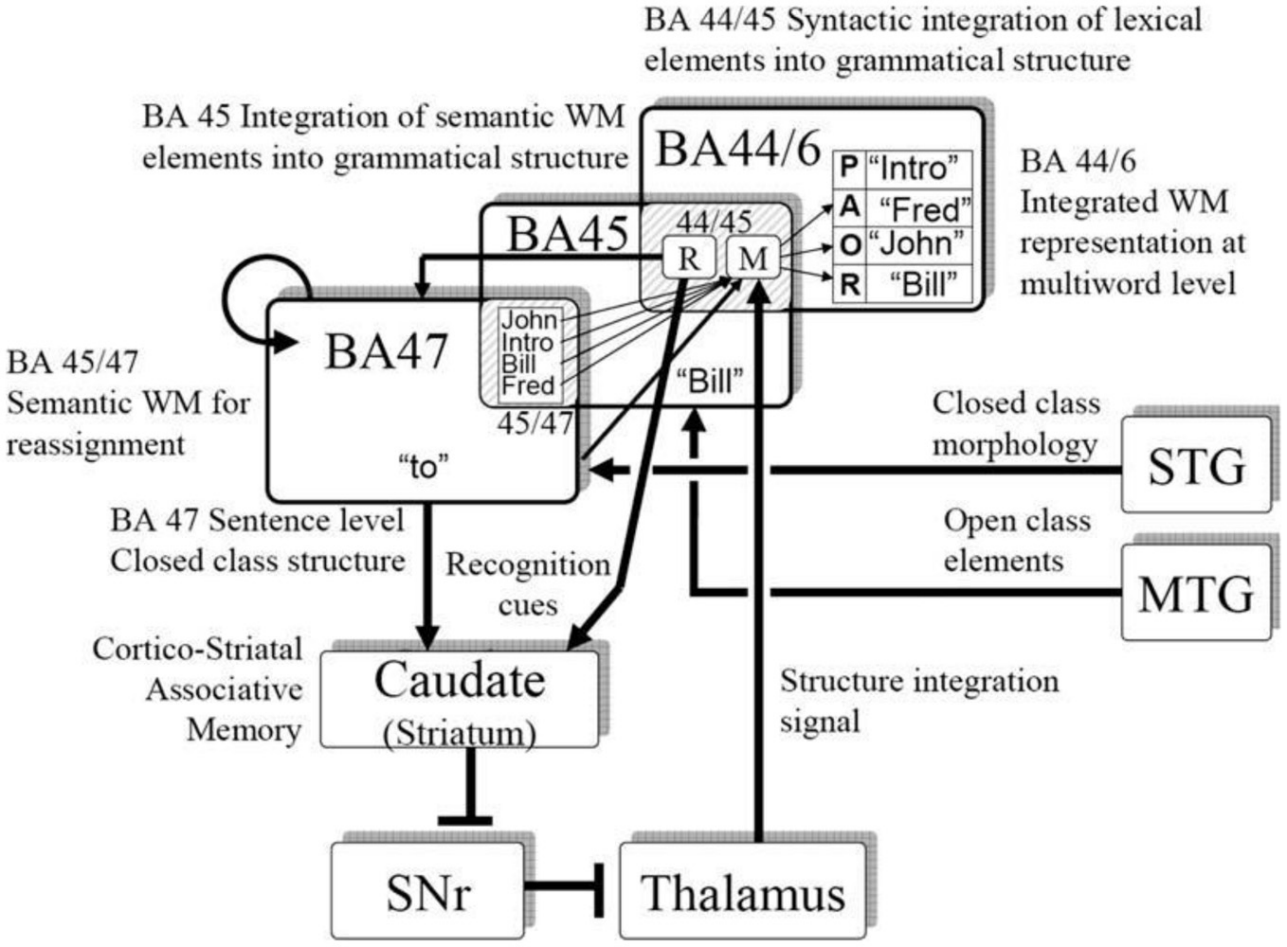
**Neurophysiologically based model of sentence and artificial grammar processing**.

Consistent with human neurophysiology, a central premise in our modeling is that the pattern of grammatical function words is represented in a recurrent cortical network, and that via plasticity in the corticostriatal synapses, the system can learn specific constructions, including constructions in different languages. Figure 5 illustrates a mapping of the neural computations onto human brain anatomy.

We (Dominey and Inui, 2009; Dominey et al., 2009) attempted to reconcile the corticostriatal model with mainstream neurophysiological models of language processing (Friederici, 2002; Hagoort, 2005) in more detail. Lexical categorization takes place in the temporal cortex, allowing for distinct processing of grammatical function words and semantic content words. Closed class elements are processed in a recurrent frontal network (Inui et al., 2007) corresponding to BA47. The pattern of closed class words forms a characteristic representation in the recurrent network, which can become associated with the appropriate mapping of open class elements onto their respective thematic roles through corticostriatal plasticity. The resulting activity then implements this mapping via the thalamo-cortical projection to the dorsal-prefrontal area BA44/6. Thus, in the inferior frontal gyrus, we consider a transition from syntactic integration in BA47, word level semantics in BA45, and sentence level integration in BA44/6, which is to a certain extent consistent with a similar gradient of processing in the model proposed by Hagoort (2005). This allocation of brain functions to the neuroanatomical regions in Figure 5 should be considered as tentative, and potentially could be replaced by different allocation of functions. The more solid proposal and contribution of this work is the demonstration that a recurrent cortical network, likely in Broca's region, can integrate multiple cues (here word order and closed class structure) consistent with the cue-competition model, and through corticostriatal plasticity this representation can implement grammatical constructions as mappings from sentence structure to meaning, consistent with the emerging role of the corticostriatal system in language processing.

This perspective is consistent with an emerging view of a dorsal-ventral distinction in language processing. While there is indeed significant variability in the details of the functional significance of the dorsal vs. ventral streams in language, there is an emerging consensus that these streams indeed have distinct roles, with the ventral stream related to semantic and conceptual content, and the dorsal stream related to more structural aspects of language (Hickok and Poeppel, 2004; Friederici, 2012; Bornkessel-Schlesewsky and Schlesewsky, 2013). Hickok and Poeppel (2004) thus suggested that the ventral stream would account for the sound-meaning interface, and the dorsal stream would accommodate the auditory-motor interface. In Bornkessel-Schlesewsky and Schlesewsky's model, the ventral stream is more associated with conceptual representations, and the dorsal stream is related to syntactic structuring and the linkage to action. Friederici (2012) proposes a dorsal-ventral model with the ventral stream subserving semantic integration and dorsal stream subserving structural processing. Interaction in ventral circuits linking BA45 and STG/MTG mediates semantic processing, whereas assignment of grammatical relations is mediated by dorsal connections between BA44 and STG/STS. This is consistent with the dorsal-ventral distinction in our model illustrated in Figure 5. Indeed, we noted (Dominey and Hoen, 2006) that BA44/46 can be considered to represent the frontal terminus of the dorsal visual pathway, and BA45 the frontal terminus of the ventral pathway (Ungerleider et al., 1998). In this neurophysiological context, Friederici (2012) points out the need to better understand the role of subcortical structures, including the striatum, in language processing. We suggest that corticostriatal plasticity plays a role in implementing the structural mapping processes required for assignment of open-class elements to their appropriate thematic roles. Ullman notes that this is consistent with his declarative-procedural model of language processing, in which the cortico-striatal system contributes to the procedural learning of grammatical rules (Ullman, 2004).

## Larger corpora and generalization in the reservoir computing framework

One of the major limitations with the neural implementation of our model of corticostriatal function is related to the performance of the learning algorithm. A simple form of reward based learning is used to associate states of activity in the recurrent network with neurons in the striatum that correspond to the appropriate thematic role assignment. This requires repetitive training on the corpus with progressive adjustment of learning rates which is prohibitive for the investigation of large corpora. In order to resolve this problem, we apply more robust machine learning methods to our corticostriatal model, in the context of reservoir computing. In reservoir computing, a reservoir of neurons with fixed recurrent connections is stimulated by external inputs, and the desired output is produced by training connections from the excited reservoir units and readout neurons. As noted in Pascanu and Jaeger (2011) this reservoir principle was independently discovered in our own work in cognitive neuroscience with the TRN (Dominey et al., 1995), in computational neuroscience (Maass et al., 2002) with the liquid state machine of Maass, and in machine learning (Jaeger, 2001) with the echo state machine of Jaeger. In the machine learning domain, fast and efficient mechanisms for learning the reservoir-to-readout connections have been developed, and this provides a significant improvement in performance for sentence processing. Using these methods, rather than repeated training with multiple iterations through the corpus, we could present the corpus to the reservoir only once, collect the reservoir activation and then use linear regression to learn the connections between reservoir units and readout units coding the meaning of the sentences.

**Figure 6:**
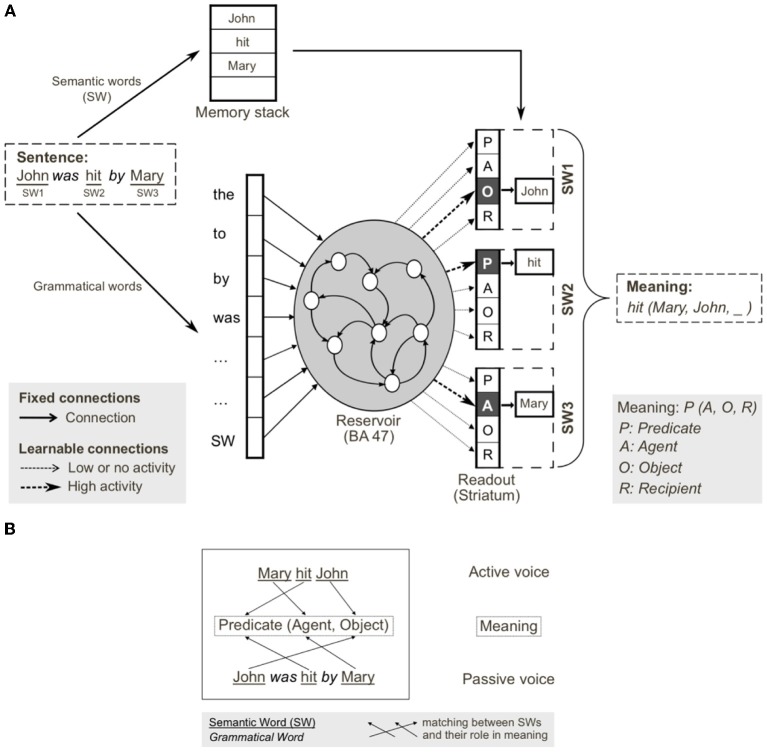
**Reservoir computing implementation of the cortico-striatal sentence processing model. (A)** Semantic and grammatical words (i.e., open and closed class words, respectively) are separated on input. Semantic words (SW) are stored in a memory stack. Grammatical words and a single input for all SWs are inputs to the reservoir (analogous to prefrontal cortex). During training, input sentences are presented word-by-word, and readout units (corresponding to striatum) are forced to the corresponding coded meaning (i.e., SW1-Object, SW2-Predicate, SW3-Agent). In testing, readout units code the predicted role(s) of each semantic word, forming the coded meaning. The meaning [i.e., hit(Mary, John, _)] can be reconstructed from the coded meaning, as SWs in memory stack are reassigned to the thematic roles (predicate, agent, object, recipient) identified in the read-outs. **(B)** Active and passive grammatical constructions (i.e., mapping from sentence form to meaning), and their shared meaning. Coded meaning (indicated by the arrows) corresponds to specific mapping from open class words to meaning, which defines the grammatical construction. From Hinaut and Dominey (2013).

**Figure 7:**
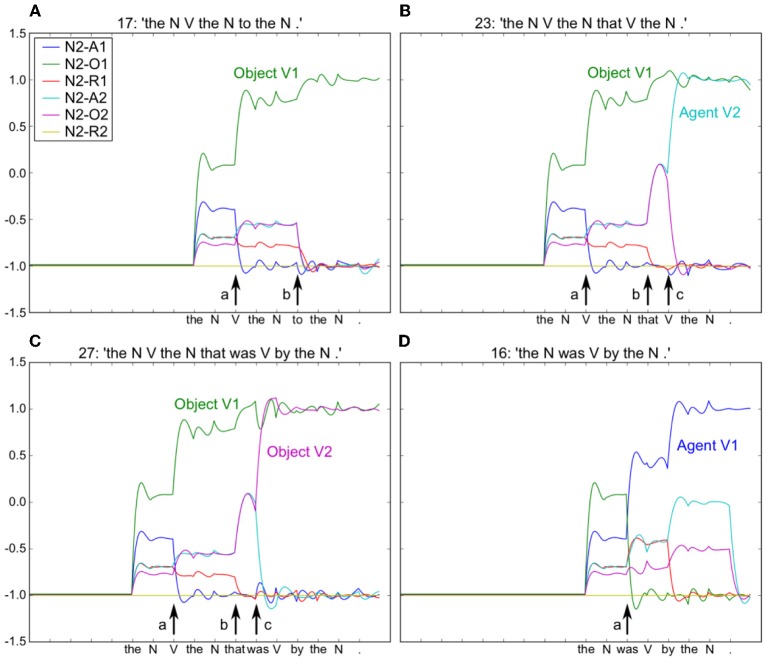
**Neurons coding thematic roles indicated by colored traces (see embedded legend).** For all four sentences [see period before arrow (a)], the model initially predicts that Noun 2 is the Object of Action 1 (green trace). In **(B)** and **(C)** this remains true, but Noun 2 is also the Agent and Object of Action 2 in **(B)** and **(C)** respectively. At point (b), arrival of “to” confirms the correct prediction of N2-O1 (green trace) in **(A)**, and the arrival of “that” induces a change in activity in **(B)** and **(C)**, with increased prediction of both Agent and Object roles for V2, respectively. Note that this is resolved at the arrival of the “V” and “was” in **(B)** and **(C)** respectively [arrow (c)]. In **(D)** the arrival of “was” provokes a new analysis with Noun 2 as the Agent of Action 1. Embedded legend: N2-A1 – Noun 2 is the agent of Action 1. A, Agent; O, Object; R, Recipient.

Using the model in Figure 6, we provided input sentences one word at time, with grammatical words feeding into the recurrent reservoir. Starting at the outset of the sentence presentation, the corresponding readout neurons that coded the correct role for each semantic word, were activated. The model was trained to generate these activations starting at the outset of the sentence, thus providing for a potential predictive capability. This training protocol corresponds to the infant seeing and interpreting the scene before hearing the sentence. Figure 7 illustrates the activation of a set of readout neurons during the presentation of four different sentence types. The individual traces represent activation of illustrative readout neurons coding for the role of the second noun. We observe that from the outset of the sentence presentation, the system predicts that Noun 2 is the object of verb 1^1^. This remains true in three of the four illustrated constructions, with the exception of the passive in panel **(D)**. Note that when the word “was” arrives, the system reconfigures its prediction. Later in these constructions (at the point indicated by the labeled arrow b) note the distinct responses respectively to “to,” and “that,” and then finally at the point indicated by the labeled arrow c, the responses to the arrival of “Verb” vs. “was.” What we observe is that time locked with words that designate the possible licensing of a construction, the model neurons react. If we dissect in panels **(B,C)** what happens between the events labeled b and c, we can consider that the system is maintaining parallel parses, and the decision between these parses is determined when the appropriate disambiguating words arrive at point c. This graphically illustrates the ranked parallel parses. That is, each of the neurons in these panels corresponds to a possible role for Noun 2. Activation of these neurons corresponds to the choice, and the level of activation corresponds to the rank. Thus, multiple parses can be entertained in parallel. In panels **(B,C)**, between marked locations b and c, two possible parses are equally active, and at the arrival of the next word at c, the choice is made.

The changes in neural activation as observed at point *c* can be interpreted in the context of human brain activity, revealed by event related potentials (ERPs) recorded during sentence processing. We can consider that the summed relative changes in activity of the model neurons represent a form of ERP signal. In this context, a larger ERP response was observed for subject-object vs. subject subject relative sentences time locked with the disambiguating word in the sentence (Hinaut and Dominey, 2013), similar to the effect observed in human subjects (Friederici et al., 2001). In our corpus, similar to human language (Roland et al., 2007), constructions with subject-object structure are less frequent than subject-subject, and canonical types where the head noun is the agent. Thus, this change in neural activity is in a sense due to a form of expectation violation, based on the corpus statistics. MacDonald and Christiansen (2002) have provided detailed simulation evidence for such phenomena involving an interaction between complexity, frequency, and experience. They demonstrated that with an equal distribution of subject- and object-relatives, their recurrent network gave superior performance on the subject relatives due to the networks' abilities to generalize to rare structures as a function of experience with similar, more common simple sentences.

The performance of the model, as revealed by these readout activation profiles can potentially be linked to reading times, such that the time required for a neuron to reach a threshold could be plausibly interpreted as a reading time.

The model thus provides an implementation of a form of ranked parallel processing model, where the parallel maintenance of possible parses is an inherent aspect of the model (Gibson and Pearlmutter, 2000; Lewis, 2000). This behavior is a reflection of the statistical structure of the training corpus. In effect, the activity of the readout neurons reflects the probability of their being activated in the training corpus.

Indeed, the behavior of the trained system is clearly influenced by the nature of the grammatical structure inherent in the training corpus. Working in the machine learning context of reservoir computing allowed us to perform experiments with corpora up to 9 × 10^4^ different constructions. The advantage of performing these large corpora experiments is that it allows for a systematic analysis of the influence of the training corpus on the ability to generalize. Here we speak of compositional generalization, where the system is actually able to handle new constructions that were not used in the training corpus (as opposed to using learned constructions with new open class words). We performed a series of experiments with a small corpus of 45 constructions in which we examined very specific timing effects of the parallel processing, and two larger corpora of 462 and 90,582 distinct constructions, respectively. Training with the larger corpora revealed quite promising generalization in cross-validation studies, where different proportions of a corpus are removed from training, and then used in testing to evaluate generalization. We observed generalization of up to 90% for the 462 corpus, and over 95% in the 90 K corpus. Interestingly, when we scrambled the 462 corpus, generalization was reduced to 30%, indicating that the system learned the underlying grammatical structure encoded in the training corpus. Most interestingly, this generalization in the largest corpus could be achieved with exposure to only 12.5% of the corpus. Thus we see that the grammatical structure of language can be learned and generalized by such recurrent networks. The clear distinction in this work is that the learning is revealed by extracting thematic roles, rather than predicting the next word or lexical category (Tong et al., 2007). Indeed, the power of such recurrent models in now employed in concrete natural language processing applications of semantic role labeling (Barnickel et al., 2009).

## Discussion

The study of recurrent networks for language processing has a rich history. A vast and productive line of research has characterized language processing in terms of predicting the next word in a sentence, initiated by the ground-breaking work of Elman (1990, 1991, 1993), and fruitfully pursued by others (e.g., Christiansen and Chater, 1999). Characterizing language in terms of thematic role assignment also has a rich history in connectionist modeling (e.g., McClelland et al., 1989; Miikkulainen, 1996). Miikkulainen provides an extensive review of this literature. His novel contribution is a modular distributed architecture based on the separation of parsing (an SRN), segmentation (a RAAM model), and a stack (for handling depth recursion). Communication between the modules includes the transfer of activation patterns, and control. The resulting system can learn case role mapping, as well as phrasal embedding structure, and then generalize these to sentences with new relative phrase embedding structure.

The research reviewed here presents a coherent framework in which a recurrent network encodes grammatical structure in the input, and modifiable connections from the recurrent network learn the mappings from that grammatical structure to the corresponding meaning representations for large corpora of grammatical constructions. With sufficiently large corpora the system displays a significant capability to generalize to new constructions, exploiting the regularities that define grammatical well formedness (Hinaut and Dominey, 2013). This argues that a system can display the ability to learn the grammatical structure implicit in a corpus without explicitly representing the grammar (Elman, 1991), and that it can generalize to accommodate new constructions that are consistent with that grammar (Voegtlin and Dominey, 2005). Part of the novelty in the position suggested here is that this recurrent network and readout system is implemented in the primate brain in the cortico-striatal system.

The computational properties of such recurrent systems is remarkable. Our initial work with recurrent networks with fixed connections and modifiable readouts demonstrated significant sequence learning capabilities (Dominey, 1995, 1998a,b), and accounted for neural coding of sequential structure in the PFC (Dominey et al., 1995; Dominey and Boussaoud, 1997). Subsequent work with such systems demonstrated their vast computational power (Maass et al., 2002; Jaeger and Haas, 2004). Projecting inputs into such reservoirs allows a mapping into a high dimensional space. This provides a dynamic compositionality that can represent an arbitrary class of non-linear functions. Recent studies of primate electrophysiology provide evidence that indeed, the PFC operates based on reservoir-like properties (Rigotti et al., 2010, 2013). The key point—the use of random connection weights in a structured network—is echoed in the principal property of cortex—the high predominance of local recurrent connectivity (Douglas et al., 1995; Binzegger et al., 2009), particularly in PFC (Goldman-Rakic, 1987). The use of fixed recurrent connections within these reservoirs means eliminates the need to artificially constrain the processing of time and temporal structure in these networks, thus allowing a realistic processing of temporal structure that is much more difficult in networks with learning in the recurrent connections. Of course, there is plasticity in the cortex, but simplifying this with fixed connections, the dynamic compositionality of reservoir computing yields significant processing capabilities. The reservoir framework is thus highly appropriate for the study of complex cognitive function including establishing structure-meaning relations in language, and it has already been successfully employed in the context of predicting the next word in the context of language processing (Tong et al., 2007).

It should be noted that dynamic does not correspond to “out of control.” That is, while a recurrent network will evolve in a dynamic pattern of activity, this dynamic activity can be associated with a stable representation of the meaning. In the human, this dynamic activity is observed in EEG responses (e.g., the ELAN, LAN, N400, P600 cascade of responses, modulated by lexical category and sentence context) that are dynamic in time, yet reflect the coherent processing of the successive words in sentences.

We have postulated that recurrent cortical networks provide the mechanism for representing grammatical structure, and that plastic corticostriatal connections participate in learning this structure in the acquisition of a language (Dominey and Inui, 2009; Dominey et al., 2009). We thus take a strong stance on the role of the human corticostriatal system in language processing. This would predict that patients with dysfunction in the corticostriatal system should have deficits in syntactic processing, and should show neurophysiological anomalies during language processing. Significant data from a number of sources are consistent with this stance. Several studies from Friederici and Kotz (2003; Friederici et al., 2003; Frisch et al., 2003; Kotz et al., 2003) in patients with striatal dysfunction due to lesion or Parkinson's disease demonstrate that the P600 ERP evoked by syntactic anomalies or complexity in normal controls subjects is reduced or eliminated in these patients, while other language related responses (including the early left anterior negativity or ELAN and N400) remain intact. Similarly, these patients are impaired in the processing of grammatical complexity (Hochstadt et al., 2006; Hochstadt, 2009). This suggests that the intact corticostriatal system is required in generating this normal brain response to grammatical complexity processing. Ullman argues that the corticostriatal system implements procedural rules for word level grammatical processing (Ullman, 2001). We take this suggestion even further, arguing that the corticostriatal system participates in the implementation of grammatical constructions at the sentence level, in the mapping of the structure of the surface form of the construction to the meaning representation (Dominey and Inui, 2009; Dominey et al., 2009).

It is now relatively accepted that meaning is encoded in distributed embodied representations that have an analogical component that is not symbolic (Bergen and Chang, 2005; Barsalou, 2009; Lallée et al., 2010b; Madden et al., 2010). Interestingly, such representations are difficult to manipulate, when compared with symbolic representations. In this context, there is an emerging perspective that the complete language system likely involves both symbolic and distributed-embodied representations (Bergen and Chang, 2005; Barsalou, 2009; Lallée et al., 2010b; Madden et al., 2010).

This poses the question of how the link is made between language and embodied simulations. A promising area where these issues can be investigated is in the development of cognitive systems for robots. This link between language and meaning in cognitive science is not new. At the height of the cognitive revolution, Feldman and colleagues proposed the problem of miniature language acquisition as a “touchstone” for cognitive science (Feldman et al., 1990). A machine should be trained on a set of <sentence, picture> pairs, and then in testing should be able to say whether a given novel sentence correctly described a novel picture. In order to address this we modified the problem such that the training data were <sentence, video-scene> pairs. Based on the notion that infants can parse visual scenes by detecting sequences of perceptual primitives [inspired by Mandler (1999)] we developed an event parser that could detect actions including take, take-from, give, push and touch (Dominey, 2003b). Naïve subjects performed actions that were parsed by this system, and simultaneously narrated their actions, thus providing a set of <sentence, meaning> data on which to train the neural network grammatical construction model (Dominey and Boucher, 2005). The model learned a set of simple constructions and could generalize to new <sentence, meaning> pairs. We subsequently demonstrated how the system could learn to recognize such actions (Lallée et al., 2010a), similar to Siskind (2001; Fern et al., 2002). Such language—action mappings are becoming increasingly powerful in the domain of human—robot cooperation (Petit et al., 2013). What we will find, is that as the cognitive systems of robots become increasingly sophisticated, they will naturally afford richer language. For example, as mental simulation capabilities develop, the need for verb aspect to control the flow of time in these simulations will naturally arise (Madden et al., 2010).

Arguments on the learnability of language have held that because the compositional generative complexity of language is so vast, and the input to the child so impoverished, the underlying language learning capability must rely on a form of pre-specified universal grammar so that language learning consists in setting the parameters of this system (Chomsky, 1995). Usage-based approaches to acquisition argue, in contrast, that the input is actually guided by joint attention mechanisms and specialized mechanisms for human socialization which focus the learners attention on the intended meaning (Tomasello, 2000, 2003; Dominey and Dodane, 2004). This suggests that language acquisition should be characterized not formally as a problem of grammar induction, but rather socially, as a problem of expressing and extracting meaning. This perspective emphasizes the potential contribution that the structure of meaning can contribute to the learning process.

In this context, Chang (2002) has demonstrated that under equivalent conditions, providing a language processing model with a message that contained semantic content provided additional structuring information, and increased the learning performance. It is likely that this contributes to generalization. We have demonstrated that with corpora of moderate size (between 450 and 90,000 constructions) the recurrent network model demonstrates quite robust generalization (Hinaut and Dominey, 2013). We believe that this is because the structural regularities that allow the system to generalize are inherent within the training data. Interestingly, the training data include both the surface forms of the constructions, and the corresponding meaning structure. This suggests that part of what allows the system to generalize is this additional source of learnable structural regularities—not only those present in the surface structure, but also those present in the mapping of that structure to the meaning structure. Thus the meaning structure can contribute to learnability and generalization (Dominey, 2000; Chang, 2002). In response to Dominey's commentary (Dominey, 2003a) on the précis of “Foundations of Language,” Jackendoff (2003) states “In the parallel architecture it is natural to suppose that the hierarchical complexity of syntax is but a pale reflection of that in meaning, and it exists only insofar as it helps express thought more precisely. Moreover, Dominey says, access to the compositionality of meaning provides a scaffolding for the child's discovery of syntactic structure. I concur.” Thus, in the study and modeling of language acquisition, significant work remains in characterizing the structure of the conceptual system.

### Conflict of interest statement

The author declares that the research was conducted in the absence of any commercial or financial relationships that could be construed as a potential conflict of interest.

## Footnotes

^1^Hinaut and Dominey also perform a more general treatment where verbs are included in the processing of semantic (or open class words) in constructions as illustrated in Figure 6.
